# Changes in Body Composition, Energy Expenditure, and Energy Intake during Four Years of University—A Follow-Up Study

**DOI:** 10.3390/ijerph18083990

**Published:** 2021-04-10

**Authors:** Shai Olansky, Kayleigh M. Beaudry, Stacey Woods, Erin Barbour-Tuck, Kimberley L. Gammage, Panagiota Klentrou, Andrea R. Josse

**Affiliations:** 1 Department of Kinesiology, Faculty of Applied Health Sciences, Brock University, St. Catharines, ON L6L 3M7, Canada; shai.olansky@gmail.com (S.O.); sw16hl@brocku.ca (S.W.); kgammage@brocku.ca (K.L.G.); nklentrou@brocku.ca (P.K.); 2 Department of Kinesiology, University of Waterloo, Waterloo, ON N2L 3G1, Canada; km2beaudry@uwaterloo.ca; 3 School of Kinesiology and Health Science, Faculty of Health, York University, Toronto, ON M3J 1P3, Canada; e.barbourtuck@usask.ca

**Keywords:** weight gain, body fat percentage, physical activity, nutrition, university

## Abstract

*Purpose*: The transition to university is often accompanied by the adoption of negative lifestyle habits, which may result in weight and fat gain. While this has been demonstrated during 1st year, little is known about subsequent years. We investigated changes in body composition, energy expenditure, and dietary/energy intake from 1st to 4th year university. *Methods*: Thirty-eight students (14 males, 24 females) completed a lifestyle questionnaire and had their body mass, fat mass, lean body mass (LBM), and body fat percentage (%BF) measured three times: at the beginning and end of 1st year, and end of 4th year. *Results*: During 1st year, body mass, fat mass, LBM, and %BF increased (+3.2 ± 3.8 kg, +2.5 ± 3.0 kg, +0.7 ± 2.1 kg, +2.3 ± 4.9%, respectively; *p* < 0.01), while daily energy intake and expenditure decreased (−359 ± 1019 kcal·d^−1^ and −434 ± 786 kcal·d^−1^, respectively; *p* < 0.01). Between the end of 1st year and end of 4th year, body mass, LBM, and energy expenditure increased (+3.2 ± 3.8 kg, +1.3 ± 2.9 kg, +209 ± 703 kcal·d^−1^, respectively; *p* ≤ 0.05), while %BF, fat mass, and energy intake did not change. *Conclusions*: Although %BF and fat mass remained stable from the end of 1st year to the end of 4th year in this group of university students, the positive increase in energy expenditure was not enough to reverse the weight and fat gained during 1st year.

## 1. Introduction

Obesity is a global problem, with a worldwide prevalence that has almost tripled since 1975 [1,2]. In Canada, 34% of the adult population is overweight, and 27% is obese [1,2]. Obesity can lead to chronic diseases such as diabetes, stroke, heart disease, musculoskeletal disorders, and cancer [3]. Furthermore, once acquired, overweight or obese body mass tends to be chronic, as most who attempt to lose excess mass fail [4,5].

When looking at critical periods for body mass gain in youth, the transition from high school to university/college was found to be linked with a high risk for weight gain [6]. In turn, body mass gained during this time can lead to the onset of obesity and increase the risk of chronic diseases and early mortality [7,8,9]. Body mass gain and changes in body composition during the freshman year (i.e., the first year) of university have been well studied in North American and European populations [5,10]. A meta-analysis of 22 studies from North America and Europe published between 1980 and 2014, found an average gain of 3.3 kg during 1st year university [11]. Additionally, Deliens et al. [10] reported that after the first semester, Belgian students gained on average 1 kg of body mass consisting mainly of fat mass, with no changes in lean body mass (LBM). Our own ‘Transition Study’ (Ontario, Canada) demonstrated significant weight gain, increased body fat percentage (%BF), lower diet quality, and less participation in sport and physical activity during 1st year university in 301 students [12,13]. We also reported that dietary changes during 1st year university included higher consumption of sugary beverages, alcohol, and fried foods, and the consumption of healthy food such as fruits, vegetables, and high-quality proteins during this time decreased [12]. Reductions in physical activity during 1st year university included decreases in light, moderate, and vigorous activity minutes [13]. Taken together, we demonstrated that changes in diet and physical activity contributed to increased body mass and %BF during 1st year university [12,13]. 

Despite the first year being well-studied, research investigating changes in body mass and composition, as well as nutrition and physical activity levels across all four years of university is limited. A cross-sectional study from the United Kingdom examining factors leading to weight gain among past and current university students who belonged to a ‘Slimming Club’ reported that gains in various years of university were inversely associated with diet quality, a greater reliance on ready meals and fast food, and low physical activity levels [14]. While these cross-sectional associations are useful, longitudinal studies following the same students over their entire time in university may be more informative, but they are limited. One such study by Hovell et al. [15] followed 43 female university students over 36 months (three years) and found that students gained an average of 1.6 kg of body mass during the first 5 months, a further increase of 1 kg after 17 months (during 2nd year), followed by a decrease back to baseline by 29 months (during 3rd year) in university. A second study by Racette et al. [16] found a significant gain in body mass during the four years in university in both male (+4.2 ± 6.4 kg) and female (+1.7 ± 4.5 kg) students. However, body composition was not assessed in either of these studies. In contrast, Gropper et al. [17] measured body composition over four years in 131 students (42 males, 89 females), but they only had two timepoints of assessment. They reported an average gain of +5.3 kg from the beginning of 1st year to the end of 4th year, and a change in %BF that was higher in males than in females (+5.2 vs. +2.9%, respectively). Although this study did measure body composition, it did not report on factors that may explain or be responsible for body mass or fat mass gains during the four years of university, such as energy intake, energy expenditure, and physical activity behaviors.

Given this gap in the literature, the present follow-up investigation assessed the changes in these variables (body mass, fat mass, LBM, %BF, daily energy intake, macronutrient intake, energy expenditure, and physical activity) at three timepoints; beginning, and end of 1st year, and end of 4th year university in a subset of participants from our original ‘Transition Study’ [12,13] that reported on the changes that occurred in body composition, nutrition, physical activity, and sport participation in 301 students during 1st year. We hypothesized that students would continue to gain body mass and %BF after 1st year, and, as such, would have higher body mass and %BF at the end of 4th year compared to the end of 1st year.

## 2. Materials and Methods 

### 2.1. Participants and Study Design 

This investigation represents a follow-up study, where we added a third timepoint of data collection, at the end of 4th year university, to the ‘Transition Study’ [12,13] that was primarily designed to investigate changes in lifestyle habits during 1st year university. Participants of the original ‘Transition Study’ were males and females, 17–20 years old, starting their 1st year at Brock University in Ontario, Canada, without any previous college or university experience. Written informed consent was obtained directly from all participants. Both the original ‘Transition Study’ [12,13] and the present follow-up study received ethical clearance from our University’s Research Ethics Board (REB# 13-297 and REB# 17-334, respectively).

Of the 301 ‘Transition Study’ participants (71 male, 230 female) who completed both the Fall and Spring visits during their 1st year of university [12,13], 38 participants (21.2 ± 0.4 years) agreed to return for the third study visit during the last (Spring) term of their 4th year. In terms of how we contacted participants for the third/final study visit, we reached out via email only to participants who agreed to be contacted for future research following the first ‘Transition Study’. Those that responded to our emails were invited back to the laboratory for testing. The reasons for the high attrition rate are unknown. Of these 38 participants who returned for the third visit, 63% were female (*n* = 24) and 37% were male (*n* = 14). Seventy-four percent of the students lived in a student house off campus (*n* = 28), 21% of the students lived at home (*n* = 8), and 5% of the students lived in residence (*n* = 2). Of the sample, 79% were Caucasian (*n* = 30), 13% were African American (*n* = 5), 3% were Arab (*n* = 1), 3% were Spanish (*n* = 1), and 2% were Asian (*n* = 1). In addition, participating students came from a variety of programs across the Faculties of Applied Health Sciences (*n* = 17), Business (*n* = 3), Education (*n* = 2), Mathematics and Science (*n* = 5), and Social Sciences (*n* = 11).

**Figure 1:**
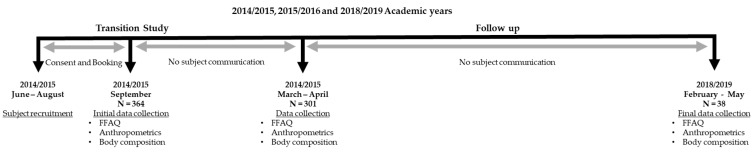
Study design.

As displayed in Figure 1, participant recruitment for the initial ‘Transition Study’ [12,13] occurred over two years during the summers (June–September) of 2014 and 2015 at Brock University. A total of 301 participants completed both the Fall and Spring study visits in their 1st year. Three years later, during the Springs of 2018 and 2019 (February–May), 38 participants returned for the follow-up study. All visits were scheduled during the hours of 8:00 a.m.–11:30 a.m. Upon arrival to the lab, participants started by answering the questionnaires. One questionnaire collected information about their general health and demographics and the other was a Food Frequency and Activity Questionnaire (FFAQ; [18]). These were also used previously [12,13]. After completing the questionnaires, the participants had their anthropometric measurements taken including height and body mass and had their body composition measured.

### 2.2. Measurements

Participants were instructed to arrive at the laboratory in a fasted state (no food or drink for 8 h prior). They were also asked to refrain from alcohol consumption for a minimum of 24 h prior and to refrain from exercise for a minimum of 12 h prior to their lab visit. These guidelines were the same as in the previous investigation [12] and were set to minimize the effects of fluid shifts and hydration status on the body composition assessment. Upon arrival to the laboratory, the participants consumed 500 mL of water. After 30 min they were asked to void their bladder just before the assessment of body mass, %BF, LBM, and fat mass using the InBody520 bioelectrical impedance analysis (BIA) system (Biospace Co. Inc., Los Angeles, CA, USA). Prior to each assessment, the electrodes were thoroughly cleaned with wipes. Participants were then instructed to stand fully upright with their arms extended not touching the sides of their body, and to refrain from moving or talking until the assessment was complete. The total time per assessment was 5 min. The InBody520 is an 8-point tactile electrode system using direct segmental multi-frequency bioelectrical impedance analysis (MfBIA). MfBIA measures resistance and reactance as a weak multi-frequency electrical current travels throughout the body. The InBody520 measures both the intracellular water and extracellular water through varying high and low frequencies from 1 kHz to 1 MHz. This system relies on empirical equations to calculate body composition based on these factors in segments because it views the human body as five cylinders (left arm, right arm, left leg, right leg, and torso). Thus, it provides separate measurements for each ‘cylinder’ summed together for accurate measurement of total body composition. To this end, two electrodes are situated under the feet on the platform, two on the palms, and two on the thumbs; the latter four are attached to handles that extend from the device. Age, height, and sex are manually entered, and body mass is assessed by a scale positioned within the device. Proprietary equations are used to calculate fat mass, LBM, and %BF from the measures of body mass, total body water, segmental impedance, and extracellular and intracellular water. MfBIA measurements of body composition have been validated against dual-energy X-ray absorptiometry [19]. BIA devices have also shown good reliability in both males and females as indicated by strong intraclass correlation coefficients (≥0.98) and low standard errors of measurement for fat mass, LBM, and %BF in healthy young men and women [20].

Height was measured with a stadiometer (Portable Fitness Scale 140-10-7N, Rice Lake Weighing Systems, Rice Lake, WI, USA) to the nearest 0.1 cm with no shoes and light clothing. Dietary intake and habitual physical activity were assessed using the 2014 Block FFAQ (NutritionQuest, Berkeley, CA, USA). This questionnaire assessed the habitual amount and frequency of consumption of 127 different food and beverage items over the past 6 months [18]. The responses were then used to calculate daily intakes of specific nutrients (i.e., carbohydrates, fat, protein), as well as total energy intake (kcal·d^−1^). The participants were also given a standard portion size sheet to help them quantify the amounts of food that they were eating and to better aid them in answering the questions. Additionally, the FFAQ was used to collect information on the frequency and duration of light, moderate, and vigorous physical activity (LPA, MPA, and VPA, respectively) of the participants on a weekly basis to calculate energy expenditure. The physical activity portion of the questionnaire also calculated the average number of metabolic equivalent (MET) minutes of light, moderate, and vigorous physical activity that the students engaged in per day, and the average daily energy expenditure for the six months preceding the survey. From the answers provided, estimates of intensity of effort and total energy expenditure in kcal·d^−1^ units were obtained.

### 2.3. Statistical Analysis 

Descriptive statistics were used to provide information about the overall characteristics of the sample. In cases where the data did not meet the assumptions of univariate normality, a log (Ln) transformation, or square root (sqrt) transformation was conducted. Body mass, fat mass, protein intake, and MPA were Ln transformed. Energy expenditure, LPA, VPA, and MET min were sqrt transformed. Finally, LBM, energy intake, and carbohydrate intake did not meet the assumptions for univariate normality, so nonparametric analyses were applied. For all data, no outliers were removed or manipulated. 

Differences in anthropometrics, body composition, physical activity, and dietary macronutrient and energy intake from the beginning of 1st year university, end of 1st year university, and end of 4th year university were examined using a two-way repeated measure analysis of variance (RM ANOVA) with sex as the between-subject variable and time as the within-subject variable. In cases with a significant main effect for time, post hoc pairwise analyses were conducted using a paired t-test. For variables showing a time-by-sex interaction, a follow-up analysis within each sex was conducted using a one-way RM ANOVA. For variables that did not meet the assumption of normality, we used a nonparametric repeated measures Friedman analysis, with Wilcoxon and Mann–Whitney U post hoc tests when needed. For all analyses, the acceptable level of significance was set at *p* ≤ 0.05. Effect sizes were also calculated, including partial eta squared (_p_*η*^2^) for ANOVA and Cohen’s d for significant pairwise comparisons [21]. Effect sizes were then interpreted based on the following criteria: 0.01 = small, 0.06 = medium, 0.14 = large effect for _p_*η*^2^, and 0.2 = small, 0.5 = medium, 0.8 = large effect for Cohen’s d [22]. The effect sizes for the Wilcoxon non-parametric tests were determined using the matched-pairs rank-biserial correlation coefficient r [23]. This r was then interpreted based on Cohen’s d conversion to the common language effect size statistics [24]: 0.56 = small, 0.64 = medium, and 0.71 = large effect. 

Finally, we used G*Power (version 3.1) to calculate the sample size *a posteriori*. Using body mass as the primary outcome, we found that for a large effect size (i.e., _p_*η*^2^ = 0.14), and *p* = 0.05, a sample size of 38 participants was sufficient to reach a power of (1 − β) = 0.8. Likewise, for the post hoc analyses, considering the following design specifications: *p* = 0.05; (1 − β) = 0.8; medium d = 0.5, the calculated sample size was also 38, but with 19 in each sex group, which is different than the number of female and male participants in our sample (24 and 14, respectively).

## 3. Results

**Figure 2:**
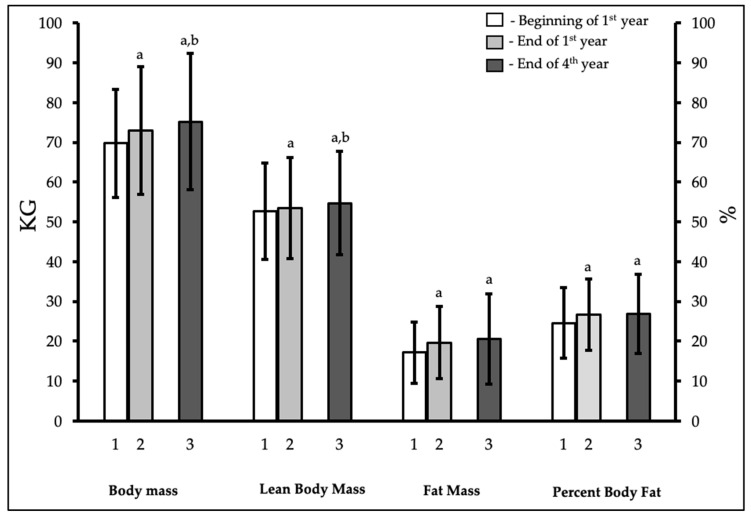
Body mass, lean body mass, fat mass, and body fat percentage levels (mean ± standard deviation) in undergraduate university students at the beginning of 1st year (1), the end of 1st year (2), and the end of 4th year (3). Notes: a, denotes significant difference from the beginning of 1st year (*p* ≤ 0.05); b, denotes significant difference from the end of 1st year to the end of 4th year (*p* ≤ 0.05) in pairwise post hoc comparisons.

Figure 2 displays the body mass and body composition results for all three timepoints, beginning of 1st year, end of 1st year, and end of 4th year university. For body mass, there were significant main effects for sex (F = 30.5, *p* < 0.001, _p_*η*^2^ = 0.46) and time (F = 15.43, *p* < 0.001, _p_*η*^2^ = 0.3), with no interaction. Significant main effects for sex (F = 17.24, *p* < 0.001, _p_*η*^2^ = 0.32) and time (F = 6.56, *p* = 0.002, _p_*η*^2^ = 0.15), with no interaction were also observed for %BF. Fat mass showed a significant main effect for time (F = 12.11, *p* ≤ 0.001, _p_*η*^2^ = 0.25), but no sex effect and no interaction. Following this, the post hoc analysis revealed that males had consistently higher body mass and lower %BF and fat mass compared to females (sex effect). Since there were no interactions, body mass, %BF, and fat mass significantly increased (time effect) from the beginning to the end of 1st year in both sexes (i.e., data combined). The average increases with the corresponding 95% confidence intervals and effect sizes were +3.2 ± 3.8 kg (+2.0 to +4.5 kg; *p* < 0.001; d = 0.84) for body mass, +2.1 ± 2.8% (+1.1 to +3.0%; *p* < 0.001; d = 0.75) for %BF, and +2.5 ± 3.0 kg (+1.5 to +3.5 kg; *p* < 0.001; d = 0.83) for fat mass. In addition, there was a significant increase in body mass from the end of 1st year to the end of 4th year (+2.2 ± 7.8 kg [+0.4 to +4.8 kg]; *p* = 0.05; d = 0.28), with no significant change in %BF (+0.2 ± 4.8% [−1.3 to +1.7%]; *p* = 0.80; d = 0.04) or fat mass (+0.9 ± 6.1 kg [−1.1 to +2.9 kg]; *p* = 0.36; d = 0.15). LBM was analyzed using a nonparametric analysis and showed a significant time effect (*χ*^2^ = 4.57, *p* = 0.033), reflecting an increase from the beginning to the end of 1st year (+0.7 ± 2.1 kg [+0.04 to +1.4 kg]; *p* = 0.039; r = 0.39) and a further increase from the end of 1st year to the end of 4th year (+1.3 ± 2.9 kg [+0.35 to +2.25 kg]; *p* = 0.011; r = 0.48) in both sexes. LBM was higher in males across all timepoints. Additionally, all body composition variables were significantly higher at the end of 4th year compared to the beginning of 1st year.

Table 1 displays the energy and macronutrient intakes from the beginning of 1st year to both the end of 1st year, and the end of 4th year university in our sample. Energy intake (kcal·d^−1^) was not analyzed for sex-by-time interactions due to the nonparametric analysis. There was a significant time effect (χ^2^ = 7.00, *p* = 0.030), reflecting a reduction in total energy intake (−17%; −359 ± 1019 kcal·d^−1^ [−694 to −24 kcal·d^−1^]; *p* = 0.002; r = 0.56) from the beginning to the end of 1st year. However, no significant changes were observed in energy intake from the end of 1st year to the end of 4th year, so by the end of 4th year, energy intake was still lower (−16%, −355 ± 895 kcal·d^−1^ [−649 to −61 kcal·d^−1^]; *p* = 0.029; r = 0.40) than it was at the beginning of 1st year (Table 1). In addition, absolute energy intake was consistently higher in males than females (*p* < 0.01). However, after adjusting for body mass, the difference in energy intake between males and females was only significant at the beginning of 1st year (40.5 vs. 27.4 kcal·kg^−1.^d^−1^, respectively; *p* = 0.014). For fat intake, there were significant main effects for both time (F = 4.36; *p* = 0.016; _p_*η*^2^ = 0.11) and sex (F = 32.22; *p* < 0.001; _p_*η*^2^ = 0.47), and a significant sex-by-time interaction (F = 3.05; *p* = 0.05; _p_*η*^2^ = 0.08). Specifically, fat consumption significantly decreased over time in males (F = 5.34; *p* = 0.038; _p_*η*^2^ = 0.29) but did not change in females. For protein intake, there was no significant sex-by-time interaction; however, a significant main effect for time was observed (F = 6.98; *p* < 0.05; _p_*η*^2^ = 0.28), reflecting a significant decrease in protein consumption from the beginning of 1st year to the end of 1st year (21%; −19.9 ± 48.8 g·d^−1^ [−36.0 to −3.9 g·d^−1^]; *p* = 0.016; d = 0.41) with no significant change from the end of 1st year to the end of 4th year. However, protein intake continued to decrease and at the end of 4th year and was 23% (−21.2 ± 46.3 g·d^−1^ [−36.5 to −6.0 g·d^−1^]; *p* = 0.008; d = 0.46) lower than the beginning of 1st year. Finally, a significant time effect was observed (χ^2^ = 14.10, *p* = 0.001) for carbohydrate intake, reflecting a decrease from the beginning of 1st year to the end of 1st year (−17%; −44.7 ± 125.7 g·d^−1^ [−86.1 to −3.4 g·d^−1^]; *p* = 0.001, r = 0.64), with no change from the end of 1st year to the end of 4th year. Yet, intake was still 18% (−46.5 ± 105.5 g·d^−1^ [−33.3 to +29.8 g·d^−1^]; *p* = 0.018; r = 0.44) lower at the end of 4th year compared with the beginning of 1st year. In addition, carbohydrate consumption was higher in males than females at all timepoints (*p* < 0.001), which was related to the higher overall intake of energy in males. However, this difference was no longer significant after adjusting for body mass. 

Table 2 displays total energy expenditure, MET min, and time spent doing physical activity broken down into categories of intensity (LPA, MPA, and VPA [in min·d^−1^]). The data are displayed for both males and females at the beginning of 1st year, the end of 1st year, and the end of 4th year university. For total energy expenditure, LPA, MPA, VPA, and MET min, there were no significant sex-by-time interactions. However, significant main effects for time were observed for total energy expenditure (F = 7.44; *p* = 0.01; _p_*η*^2^ = 0.17), LPA (F = 8.85; *p* = 0.001; _p_*η*^2^ = 0.34), MPA (F = 8.13; *p* = 0.007; _p_*η*^2^ = 0.18), and MET min (F = 9.7; *p* < 0.001; _p_*η*^2^ = 0.21). Specifically, total energy expenditure decreased from the beginning of 1st year to the end of 1st year (−38%; −434 ± 786 kcal·d^−1^ [−693 to −176 kcal·d^−1^]; *p* < 0.001; d = 0.55), followed by an increase from the end of 1st year to the end of 4th year (+29%; +209 ± 703 kcal·d^−1^ [−22.5 to +439.7 kcal·d^−1^]; *p* = 0.007; d = 0.30) in both sexes. Interestingly, although the total energy expenditure was still 20% lower at the end of 4th year compared to the beginning of 1st year, this difference was not significant. LPA followed the same pattern of decreasing during 1st year (−54%; −60 ± 100 min·d^−1^ [−93.5 to −27.6 min·d^−1^]; *p* < 0.001; d = 0.60) and increasing from the end of 1st year to the end of 4th year (+64%; +33 ± 62 min·d^−1^ [+12.8 to +53.7 min·d^−1^]; *p* < 0.001; d = 0.53). MPA decreased significantly during 1st year (−57%; −43 ± 78 min·d^−1^ [−68.5 to −17.2 min·d^−1^]; *p* = 0.004; d = 0.55), did not change from the end of 1st year to the end of 4th year, but remained lower than at the beginning of 1st year (−52%; −39.2 ± 77.0 min·d^−1^ [−64.6 to −13.9 min·d^−1^]; *p* = 0.002; d = 0.51). The MET min showed a decrease from the beginning of 1st year to the end of 1st year (−44%; −407 ± 599 min·d^−1^ [−604 to −210 min·d^−1^]; *p* < 0.001; d = 0.68); and while it increased in both sexes from the end of 1st year to the end of 4th year (+31%; +164 ± 488 min·d^−1^ [+4 to +325 min·d^−1^]; *p* = 0.007; d = 0.34), it was still lower than the beginning of 1st year (−26%; −243.2 ± 628.4 min·d^−1^ [−449.7 to −36.6 min·d^−1^]; *p* = 0.26; d = 0.39). No significant main effect for time was observed for VPA. Additionally, total energy expenditure and VPA showed a significant main effect for sex, reflecting that both were consistently higher in males compared to females across all timepoints.

## 4. Discussion

This follow-up study, in a Canadian university sample, provides important insights regarding the trajectories of change of body composition, energy intake, and energy expenditure during the undergraduate university years. We present here, two main findings. First, after a significant increase in body mass during 1st year university, body mass continued to increase from the end of 1st year to the end of 4th year, but the amount of weight gained during this latter period was less than that gained during 1st year. Moreover, the weight gained during the latter three years of university consisted of more LBM (compared to fat mass), and the %BF gained during 1st year remained stable to the end of 4th year. Thus, these students were unable to lose the fat mass (and %BF) they gained during their initial year of university, which contributed to a greater body mass after four years compared to baseline. Secondly, both daily energy intake and energy expenditure decreased during 1st year with no further change in the total energy intake but increases in energy expenditure were observed from the end of 1st year to the end of 4th year indicating an increase in physical activity during this time. 

Overall, our results suggest that lifestyle changes in university can increase the risk of students becoming overweight or obese. Indeed, by the end of the four years spent in university, the obesity rate in the current sample increased by 6% using %BF cutoffs from the World Health Organization (≥25% for men, ≥35% for women) [25]. This sheds light on the need for interventions to help students adopt healthier lifestyles specifically during 1st year university as this is the time when students undergo the greatest changes and adjustments to university life. According to our results, our participants were also unable to lose the fat mass gained in 1st year and this may have a lasting impact on their health. Thus, promoting positive, healthy habits during 1st year is recommended as it is harder to lose excess weight than it is to prevent it from being gained [4,5]. Research shows that students do gain body mass and %BF in the university years [6,17,26], yet there is a paucity of research on the time course of these gains. Studies in this area have focused mainly on the transition from high school to 1st year university [6,11]. For example, a meta-analysis of 22 studies (4 countries in North America and Europe, 5549 students) published between 1980 and 2014, found that students gained, on average, 3.3 kg during 1st year university [11]. Longitudinal studies spanning all four years of university have typically included two timepoints of data collection: at the beginning of 1st year and the end of 4th year, without any midpoint data collection [16,17]. The current study focused specifically on potential changes in body mass and body composition from the beginning of 1st year to the end of 1st year, and from the end of 1st year to the end of 4th year. Thus, we were able to capture the different trajectories of body mass and composition change while isolating the effect of 1st year. Interestingly, if we just reported data from the start of 1st year to the end of 4th year, our results would tell another story. 

In this analysis, body mass and body composition variables showed no sex-by-time interactions, and, as such, males and females were combined into one group. Importantly, students gained less weight from the end of 1st year to the end of 4th year than during 1st year university. Furthermore, although during 1st year, the students gained 2.3% body fat there were no further significant changes in %BF from the end of 1st year to the end of 4th year. Instead, during this time, there was a concomitant significant increase in LBM. Therefore, the composition of weight that was gained in 1st year was different than the composition of weight gained after 1st year. The former was characterized by greater fat mass and the latter by greater LBM. Our results from 1st year agree with results from a Belgian study showing that, during the 1st semester in university, students gained on average 1 kg of body mass consisting of mostly (0.8 kg) fat mass, with no changes in LBM [10]. Of note, the differences in the composition of weight gained across our different timepoints may relate to maturation, despite the older age of our participants (i.e., towards the end of pubertal maturation). In 1st year, most of our participants were in late adolescence (17/18 years at the start and 18/19 years at the end of 1st year), while by the end of 4th year, all participants reached adulthood (21/22 years). This difference in age and maturation throughout university may have influenced our findings of no changes in %BF and fat mass, as well as increases in LBM in the latter years as it has been previously suggested that growth during the adolescent period influences body composition measurements [27,28]. 

Previous studies investigating changes in body mass and body composition after 1st year university are limited and inconsistent. For instance, Racette et al. [16] found a significant gain in body mass during the four years in university, such that females gained 1.7 ± 4.5 kg and males gained 4.2 ± 6.4 kg. However, they did not assess body composition, so one cannot decipher whether the weight gain was due to the addition of fat mass or LBM. Likewise, they only had two timepoints of assessment, so it is not possible to determine when, over the four years, the bulk of the weight was gained. Another study by Gropper et al. [17] showed that females increased their %BF by 2.9 ± 3.2% and males by 5.2 ± 3.6% from the beginning of 1st year to the end of 4th year in university, but again, it is unclear when this weight was gained. Our study had an additional measurement after 1st year university allowing us to better understand when the weight was gained. One study by Hovell et al. [15], which monitored the weight changes in 43 women each year for three years of university, found that body mass gained during 1st year dropped almost to baseline by the end of the 3rd year. Our study and that of Hovell et al. [15], clearly demonstrate that 1st year university is a unique time for weight gain. Corroborating our insights, a longitudinal study by Morassut et al. [29] is currently underway at another Ontario (Canada) university to specifically assess the yearly trajectories of change in body mass and body composition, and to better characterize other lifestyle factors that influence these variables from the beginning of 1st year to the end of 4th year. The results are not yet published but they will provide additional insights regarding the changes during university. While not restricted to university students, longitudinal data from the Pediatric Bone Mineral Accrual Study conducted in Saskatchewan (Canada) in the early 2000s suggests that body fat increases slightly from 18–21 years in males and females, potential owing to parallel changes in lifestyle factors. Specifically, males gain 1–5 kg of fat mass with slight increases in both physical activity and energy intake, and females gain 0.5–2.5 kg of fat mass with a slight decrease in physical activity and no change in energy intake [30]. 

Our study involved university students in the period of emerging adulthood, which is generally from 18 to 25 years of age [31]. This is the time when individuals develop their adult lifestyle habits, including those related to health behaviors such as dietary intake (including alcohol consumption) and physical activity [14,31]. We demonstrated that dietary changes during 1st year included a decrease in total daily energy intake, protein intake, and carbohydrate intake, but no change in fat intake. These results are consistent with other studies that have used FFAQs to quantify daily energy intake and daily macronutrient consumption during 1st year university [32,33]. It is interesting that these studies and ours observed an increase in body mass despite a decrease or maintenance of total energy intake [12,32,33]. The decrease or maintenance in energy intake suggests that gains in body mass and %BF during 1st year university are more likely related to a decrease in energy expenditure, as this is the other main factor affecting energy balance and can contribute to weight and fat mass gains [34]. 

In terms of physical activity, during 1st year, daily energy expenditure decreased. Specifically, activity-related changes in the current sample include a decrease in LPA and MPA, but no change in VPA. These changes in physical activity during 1st year are also consistent with the changes observed in the larger sample of the ‘Transition Study’ and another study that investigated 1st year university students [13,35]. In the cross-sectional study by Sprake et al. [14], it was also found that students who gained the most weight reported lower physical activity levels and lower diet quality irrespective of the study year. Over the latter three years of university, our follow-up study demonstrated that energy intake and macronutrient consumption did not change. However, from the end of 1st year to the end of 4th year, energy expenditure significantly increased, which may help to explain why students gained less body mass, characterized by higher LBM increases and no changes in %BF. These changes, including an increase in total energy expenditure and LPA, with no change in MPA and VPA, reflect improvements in physical activity habits after 1st year university. Furthermore, the fact that only LPA increased significantly during this time suggests that the change in health habits, and possibly also in body composition, may not have been related to more structured/planned exercise (measured as MPA and VPA), but rather to the overall increased physical activity related to activities of daily living (i.e., walking around campus, to and from campus). Hence, it is likely important to emphasize to students how these functional/habitual physical activity opportunities during the day help to maintain a healthy body mass and body composition. This notion has been explored previously by Flint et al. [36] demonstrating that individuals who made more choices to increase habitual physical activity, such as walking or biking to work and using the stairs rather than the elevator, gained less weight over time than those who did not increase their activity level in this way. Increasing these types of light activities, as found in the present study, can contribute to increased daily energy expenditure [36,37,38]. 

The main strength of our study is its longitudinal design, which allowed for a comparison of body composition and lifestyle changes observed during 1st year compared with those observed from the end of 1st year to the end of 4th year. Collecting data over several timepoints in longitudinal studies better captures the true trajectories of these variables. Along these lines, the study by Kuo et al. [39], using data from the Baltimore Longitudinal Study of Aging, demonstrated that assessing body composition with increasing age using a combination of a few measures in a linear fashion can be problematic as this approach assumes that change is constant over time when it is quite heterogenous. That is, some manifestations of aging accelerate/decelerate at different rates and may even change direction over time. Thus, as we assert, a greater number of measurement points over time would better highlight these intricate trajectories, particularly, during a specific life stage, as we have shown during the university years.

This study has some limitations. The first is that energy intake and energy expenditure are estimated by questionnaires. Although these are common practices in research studies like this one, there are more objective and accurate ways to measure these constructs, including using accelerometers and software programs/portable apps but they can be costly and/or a greater burden for participants. Second, body composition assessment can be challenging, and our results cannot be directly compared to other studies because not only is there inherent variability between different types of BIA devices (e.g., hand-to-hand, foot-to-foot, foot-to-hand, direct segmental, etc.) but also between different body composition modalities in general [40,41]. Third, our sample size is small (*n* = 38). Although retention rates for longitudinal studies in university populations tend to be low, we acknowledge that the high attrition rate of the follow-up study is concerning. However, despite clear differences in the size of the samples, the same patterns of change were observed for the changes in body mass and body composition during 1st year in both the main ‘Transition Study’ (*n* = 301) [12] and the current sample (*n* = 38). This suggests that the current sample, albeit small, is representative of the original cohort. In addition, selection bias may have played a role in defining our sample as those who came back for follow-up might have done so because they had a lower body mass at baseline, a greater change in body composition or a greater appreciation for health-related information, which could have affected their participation; however, we found no other differences between participants and drop-outs at baseline for %BF, energy expenditure, LPA, MPA, VPA, energy intake, and macronutrient consumption. In addition, no differences were found between participants and drop-outs for the changes occurring from the beginning of 1st year to the end of 1st year, in any of the investigated variables.

## 5. Conclusions

Results from our follow-up study, in a cohort of undergraduate university students, demonstrated that %BF increased during 1st year, and that these gains were maintained thereafter until the end of 4th year. In the latter three years, despite an increase in physical activity, students were unable to reverse these negative changes in adiposity, which may have negative health implications. This is concerning because the weight that is gained early in life, especially during times of rapid lifestyle change (i.e., this transition period), can track into adulthood, leading to an increased risk of overweight and obesity in students during and after university [42]. Additionally, this study assessed diet and physical activity habits from an energy balance standpoint and observed no changes in energy or macronutrient consumption but an increase in physical activity from the end of 1st year to the end of 4th year. The positive changes in physical activity, which were characterized predominantly by increases in light activity, were congruent with the stabilization of %BF by the end of 4th year. This highlights the possible beneficial contribution of habitual physical activity to body composition during university. Given that most body composition changes occurred in 1st year university, the creation of health programs targeting 1st year students and promoting a healthy transition to university life should be a priority for post-secondary institutions. Future research in this area, such as the ongoing study by Morassut et al. [29], should include further detailed investigations into diet and physical activity habits with more timepoints, which will allow us to determine the trajectories of body mass and %BF change in more detail and their relationship with these lifestyle factors. Studies that include these factors will ultimately improve our understanding of not only the ideal time for an intervention, but also how best to intervene to maximize health benefits during university. 

## Figures and Tables

**Table 1:** Total daily energy, fat, protein, and carbohydrate consumption in undergraduate university students at the beginning of 1st year (T1), end of 1st year (T2), and end of 4th year (T3). Values are mean ± standard deviation [95% confidence intervals].

Nutrient	T1	T2	T3
Total energy intake (kcal·d^−1^)	2255 ± 1108 [1891–2619]	1896 ± 1140.9 ^a^ [1521–2271]	1900 ± 845 ^a^ [1622–2178]
Male (*n* = 14)	3200 ± 1077 * [2578–3821]	2671 ± 1506 * [1801–3540]	2439 ± 1062 * [1826–3052]
Female (*n* = 24)	1704 ± 678 [1418–1991]	1444 ± 482 ^a^ [1240–1648]	1586 ± 480 ^a^ [1383–1788]
Fat intake (g·d^−1^)	92.2 ± 47.4 [76.6–107.8]	78.8 ± 47.2 [63.2–94.3]	80.1 ± 37.0 [67.9–92.2]
Male (*n* = 14)	135.6 ± 43.1 * [110.7–160.5]	113.6 ± 57.1 * [80.6–146.6]	104.3 ± 43.3 ^a^* [79.3–129.3]
Female (*n* = 24)	66.9 ± 27.5 [55.3–78.5]	58.4 ± 23.9 ^a^ [48.3–68.5]	65.9 ± 24.0 [55.8–76.0]
Protein intake (g·d^−1^)	93.2 ± 58.2 [74.0–112.3]	73.2 ± 52.8 ^a^ [55.9–90.6]	71.9 ± 35.1 ^a^ [60.4–83.5]
Male (*n* = 14)	144.2 ± 61.9 * [108.5–180.0]	113.0 ± 68.7 * [73.3–152.8]	97.0 ± 41.2 ^a^* [73.2–120.8]
Female (*n* = 24)	63.4 ± 27.7 [51.6–75.1]	50.0 ± 16.9 ^a^ [42.9–57.1]	63.4 ± 27.7 [48.6–65.9]
Carbohydrate intake (g·d^−1^)	264.3 ± 117.1 [225.8–302.7]	219.5 ± 137.6 ^a^ [174.3–264.7]	217.8 ± 101.5 ^a^ [184.4–251.1]
Male (*n* = 14)	347.5 ± 116.5 * [280.2–414.7]	295.1 ± 194.0 * [183.1–407.1]	272.2 ± 136.6 * [193.3–351.1]
Females (*n* = 24)	215.7 ± 87.9 [178.6–252.8]	175.4 ± 60.7 ^a^ [162.3–201.1]	186.0 ± 56.2 [162.3–209.8]

^a^ denotes significant difference from T1, i.e., beginning of 1st year (*p* ≤ 0.05) in pairwise post hoc comparisons; * denotes significant (*p* ≤ 0.05) difference between males and females at a given timepoint.

**Table 2:** Total daily energy expenditure and physical activity in undergraduate university students at the beginning of 1st year (T1), end of 1st year (T2), and end of 4th year (T3). Values are mean ± standard deviation [95% confidence intervals].

	T1	T2	T3
Energy Expenditure (kcal·d^−1^)	1151 ± 691 [924–1378]	717 ± 779 ^a^ [461–973]	925 ± 626 ^b^ [720–1131]
Male (*n* = 14)	1519 ± 717 * [1105–1933]	1256 ± 1039 * [656–1376]	1246 ± 582 * [910–1582]
Female (*n* = 24)	937 ± 588 [688–1185]	402 ± 293 [278–526]	738 ± 583 [492–984]
LPA (min·d^−1^)	111.8 ± 86.2 [83.4–140.1]	51.2 ± 52.9 ^a^ [33.8–68.6]	84.5 ± 54.3 ^b^ [66.7–102.3]
Male (*n* = 14)	84.1 ± 72.0 [42.6–125.7]	64.9 ± 77.1 [20.4–109.5]	80.8 ± 40.6 [57.4–104.3]
Female (*n* = 24)	127.9 ± 91.1 [89.4–166.4]	43.2 ± 31.0 [30.2–56.3]	86.6 ± 61.6 [60.6–112.6]
MPA (min·d^−1^)	75.2 ± 70.5 [52.1–98.4]	32.4 ± 48.5 ^a^ [16.4–48.3]	36.0 ± 47.9 ^a^ [20.2–51.7]
Male (*n* = 14)	64.6 ± 58.8 [30.6–98.6]	52.3 ± 66.1 [14.1–90.5]	25.7 ± 25.5 [11.0–40.5]
Female (*n* = 24)	81.4 ± 77.0 [48.9–113.9]	20.7 ± 30.4 [7.9–33.6]	41.9 ± 56.8 [18.0–65.9]
VPA (min·d^−1^)	55.4 ± 56.3 [36.9–73.9]	40.3 ± 43.7 [26.0–54.7]	49.6 ± 45.9 [34.5–64.6]
Male (*n* = 14)	91.4 ± 65.2 * [53.8–129.1]	66.2 ± 56.5 * [33.6–98.8]	74.0 ± 41.4 * [50.0–97.9]
Females (*n* = 24)	34.3 ± 38.0 [18.3–50.3]	25.2 ± 24.8 [14.7–35.7]	35.3 ± 42.9 [17.2–53.4]
MET min	929 ± 503 [763–1094]	521 ± 481 ^a^ [363–679]	685 ± 409 ^a,b^ [551–820]
Male (*n* = 14)	1071 ± 529 [765–1376]	805 ± 635 * [438–1172]	801 ± 323 [615–988]
Females (*n* = 24)	846 ± 478 [644–1048]	356 ± 258 [246–465]	618 ± 444 [430–805]

LPA = light physical activity; MPA = moderate physical activity; VPA = vigorous physical activity. ^a^ denotes significant difference from T1, i.e., beginning of 1st year (*p* ≤ 0.05) in pairwise post hoc comparisons; ^b^ denotes significant difference from T2, i.e., end of 1st year (*p* ≤ 0.05) in pairwise post hoc comparisons. * denotes significant (*p* ≤ 0.05) difference between males and females at a given timepoint.

## Data Availability

The data presented in this study are available on request from the corresponding author [A.R.J.] for researchers who meet the criteria for access to confidential data. The data are not publicly available due to REB restrictions.
